# Psychometric Properties of a Fidelity Scale for Illness Management and Recovery

**DOI:** 10.1007/s10488-019-00992-5

**Published:** 2019-11-07

**Authors:** Karina Myhren Egeland, Kristin Sverdvik Heiervang, Matthew Landers, Torleif Ruud, Robert E. Drake, Gary R. Bond

**Affiliations:** 1 Division of Mental Health Services, Akershus University Hospital, Sykehusveien 25, 1478 Lørenskog, Norway; 2 Institute for Clinical Medicine, University of Oslo, Oslo, Norway; 3 Westat, Philadelphia, USA

**Keywords:** IMR Fidelity scale, Psychometric properties, Illness Management and Recovery, Measurement

## Abstract

This study examined the psychometric properties and feasibility of the Illness Management and Recovery (IMR) Fidelity scale. Despite widespread use of the scale, the psychometric properties have received limited attention. Trained fidelity assessors conducted assessments four times over 18 months at 11 sites implementing IMR. The IMR Fidelity scale showed excellent interrater reliability (.99), interrater item agreement (94%), internal consistency (.91–.95 at three time points), and sensitivity to change. Frequency distributions generally showed that item ratings included the entire range. The IMR Fidelity scale has excellent psychometric properties and should be used to evaluate and guide the implementation of IMR.

**Trial registration**: ClinicalTrials.gov Identifier: NCT03271242.

## Background

Evidence-based practices (EBPs) require reliable and valid instruments to assess fidelity (Bond et al. 2011; Martinez et al. 2014; McHugo et al. 2007). Fidelity to interventions, defined as the degree to which an implementer follows the intervention as specified (Cross and West 2011), is one critical implementation outcome (Proctor et al. 2011).

Illness Management and Recovery (IMR) is a standardized psychosocial intervention designed to help people with serious mental illnesses manage their illness and achieve personal recovery goals (Mueser et al. 2006). Five strategies form the basis of the IMR program: psychoeducation to improve knowledge of mental illness, relapse prevention to reduce relapses and hospitalizations, behavioural training to improve medication adherence, coping skills training to reduce the severity and distress of persistent symptoms, and social training to strengthen social support. The practitioners teach these strategies using a combination of educational, motivational, and cognitive-behavioural techniques, following an accompanying workbook with educational handouts in weekly sessions over 10–12 months, either individually or in groups. The IMR program has spread world-wide (Egeland et al. 2017; Garber-Epstein et al. 2013; Pratt et al. 2011; Roosenschoon et al. 2016), including strong endorsement in Sweden (The National Board of Health and Welfare 2017). A 2014 review concluded that IMR had superior outcomes to treatment as usual, according to observer ratings of psychiatric symptoms, as well as patient and practitioner ratings (McGuire et al. 2014).

The *Illness Management and Recovery Fidelity Scale* (IMR fidelity) (McHugo et al. 2007) assesses the implementation of specific strategies within the IMR program together with structural and curriculum-based elements, with each item rated on a behaviorally anchored continuum from 1 = no fidelity to 5 = excellent fidelity. A summed and averaged fidelity score of 4.0 or higher or more defines good fidelity, 3.0–4.0 as fair fidelity, and less than 3 as an absence of fidelity (Bond et al. 2009; McHugo et al. 2007). Psychometric assessment of the scale has been limited. One study demonstrated high inter-rater reliability (ICC = .97) (McHugo et al. 2007), and two studies found sensitivity to change following training and consultation (McHugo et al. 2007; Salyers et al. 2009). Nevertheless, no published study has reported a comprehensive psychometric assessment of the IMR Fidelity scale.

This study examined the psychometric properties of the Illness Management and Recovery (IMR) Fidelity scale, including item analysis, interrater reliability, interrater item agreement, internal consistency, and sensitivity to change.

## Method

### Overview

As part of a large implementation study (ClinicalTrials NCT03271242), the research team invited mental health clinics providing treatment for psychosis disorders throughout Norway to participate in a study of implementing evidence-based practices. Eleven sites from six of the 19 health trusts in Norway agreed to implement IMR and received intensive technical assistance in implementing IMR. The current paper reports the findings of a secondary data analysis of IMR fidelity assessments at these 11 sites. Prior to the study initiation, none of the sites were providing IMR. All sites committed to adopting IMR and following the program model and practice manual (Gingerich and Mueser 2011). The Regional committees for medical and health research ethics (REK 2015/2169) approved the study, which followed the principles in the Declaration of Helsinki.

### Study Sites

Eight of the 11 mental health clinics were community mental health centers, one was a combined inpatient and outpatient clinic for young adults with psychosis and drug abuse problems, one was an outpatient clinic for children and adolescents, and one was an inpatient clinic for adolescents. The latter sites enrolled youth aged 16 years and older in the IMR program. The participating clinics represented both urban and rural areas.

### Procedures

Each clinic received intensive technical assistance in IMR over 12 months. The technical assistance included 4 days of IMR training with a professional trainer, followed by 30-min weekly group supervision by phone for 6 months, and then every other week for another 6 months.

Each site received a fidelity assessment at baseline, and after 6, 12, and 18 months. A pair of fidelity assessors, independent from the clinical staff delivering IMR, conducted each fidelity assessment. The fidelity assessors varied across sites and assessment periods. A group of 17 researchers (psychologists, psychiatrists, nurses and other health professionals) served as the assessors. All received specific training on IMR fidelity assessment. A senior researcher served as a quality control monitor, reviewing all the fidelity assessments.

The assessors conducted full-day site visits, using an integration of four sources of information: (a) semi-structured interviews with the site leader, (b) semi-structured group interviews with the practitioners that facilitated IMR, (c) progress notes on the patients’ goals and steps towards the goals that were filled out by the practitioners prior to the site visit, and (d) handouts and written materials on the patients’ progress. The two assessors rated each program independently and then compared ratings, resolving discrepancies through discussion in order to reach consensus.

### Measures

The Illness Management and Recovery Fidelity scale (McHugo et al. 2007) assesses the implementation of specific strategies within the IMR program, such as goal setting and follow-up, motivational techniques, educational techniques, cognitive-behavioral techniques, coping skills training, relapse prevention training, and behavioral tailoring for medication. It also assesses structural and curriculum-based elements, including the number of people in a group, the number of sessions held, the content modules covered, provision of educational handouts, and involvement of significant others (see “Appendix” section). The scale consists of 13 items, with each scored on a 5-point scale (from one indicating no implementation and five indicating full implementation).

A Norwegian translation agency translated the IMR Fidelity scale into Norwegian, in conjunction with the translation of the IMR manual (Egeland 2018). Two of the authors (KME and KSH) reviewed the translation in detail, repeatedly comparing it with the original version. A prior implementation project tested the translated version (Egeland et al. 2017).

### Data Analyses

We examined agreement between assessors at the item level by percentage of exact agreement between pairs of assessors. We also examined mean agreement at each time period and across items for each of four time periods.

We calculated each assessor’s total fidelity score for each site, defined as the sum of the item ratings divided by the number of items (i.e., 13). To evaluate interrater reliability of the site fidelity ratings, we used the intraclass correlation coefficient (ICC) (McGraw and Wong 1996), based on a one-way random effects analysis of variance model for agreement between the two fidelity assessors on the IMR Fidelity scale. A single ICC was computed, combining paired ratings across all assessment points.

After assessing interrater agreement and reliability, we used consensus ratings in all subsequent analyses. To estimate internal consistency of the IMR scale, we used Cronbach’s alpha, calculating an alpha coefficient for each time period.

We next examined the item distributions at 18 months, examining mean, standard deviations, and distribution of scores across sites for full (rating = 5), adequate (4), and poor (1–3) fidelity. We also examined the distribution of site scores at 18 months.

Finally, we examined the longitudinal pattern of fidelity graphically and statistically. We examined sensitivity for change over time in IMR fidelity using a one-way ANOVA repeated measures design with pairwise post hoc tests with Bonferroni correction for changes between baseline and each of the three follow-up assessments. Change over time was estimated by calculating the standardized mean difference effect size (Cohen’s d_z_) for within-subjects design (Lakens 2013). All data analyses were done using SPSS for Windows version 25 (https://www.ibm.com/analytics/us/en/spss/spss-statistics-version/).

## Results

### Agreement Between Assessors on Individual Items

Over all items and time periods, exact agreement on items was very high, averaging 94% (see Table 2 in the “Appendix” section). The mean exact agreement declined from 99% at baseline to 90–93% thereafter. (High agreement at baseline was due to lack of IMR implementation and ratings of one.) At the item level, mean agreement on all four fidelity reviews on Item 13 (behavioural tailoring) was 82% and on Item 7 (goal follow-up) was 87%, while mean agreement on all other items exceeded 90%.

### Interrater Reliability

Two fidelity assessors rated the IMR Fidelity scales on four occasions at each of the 11 participating sites. We aggregated paired ratings across four time periods to estimate interrater reliability for the 44 assessments (100% completion rate). The intraclass correlation measuring interrater reliability (assuming two assessors) was .99, indicating a very high degree of agreement. In all subsequent analyses, we report the findings based on consensus ratings.

### Internal Consistency

After baseline, internal consistency (Cronbach’s alpha) was excellent: undefined (baseline), .91 (6 months), .94 (12 months), and .95 (18 months), suggesting that the 13 items comprising the IMR Fidelity scale are measuring a unitary construct. Internal consistency at baseline could not be calculated because nearly all items were rated 1 at all sites.

### Item Analysis

As shown in Table 1, the item means for the 11 sites at 18 months ranged from 4.18 (Item 2: Program Length and Item 5: Involvement of Significant Others) to 4.82 (Item 12: Relapse Prevention Training). Notably, all of the items reached an average score exceeding 4.0, which is the benchmark for good fidelity. By contrast, at baseline, all mean item ratings were 1.60 or less. Thus, fidelity assessors used the entire rating scale from 1 to 5 for all 13 items, with no evidence of restriction of range.

**Table 1:** Item distributions on the IMR Fidelity scale at 18 months (N = 11 sites)

	Item description	Level of fidelity (% of sites)			
		Mean (SD)	Poor (1–3) (%)	Adequate (4) (%)	Full (5) (%)
1	People in session/group	4.64 (1.21)	9	0	91
2	Program length	4.18 (1.60)	18	9	73
3	Curriculum comprehensiveness	4.36 (1.43)	18	0	82
4	Educational handouts	4.64 (1.21)	9	0	91
5	Involvement of significant others	4.18 (1.60)	18	9	73
6	IMR goal-setting	4.64 (1.21)	9	0	91
7	IMR goal follow-up	4.27 (1.27)	18	18	64
8	Motivation-based strategies	4.64 (1.21)	9	0	91
9	Educational techniques	4.64 (1.21)	9	0	91
10	Cognitive-behavioral techniques	4.55 (1.21)	9	9	82
11	Coping skills training	4.55 (.69)	9	27	64
12	Relapse prevention training	4.82 (.40)	0	18	82
13	Behavioral tailoring for medication	4.45 (.69)	9	36	55
	Total scale	4.50 (.96)	9	18	73

Items rated on a 5-point scale, with 5 = fully implemented

### Changes over Time

**Fig. 1:**
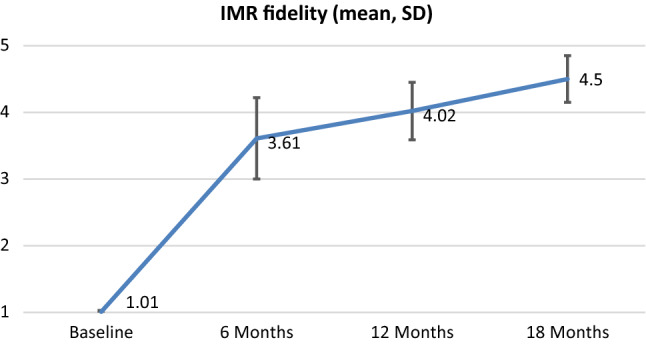
Development of IMR fidelity from baseline to 18 months

We inspected the longitudinal pattern of changes graphically across the 18-month period for the 11 sites, as shown in Fig. 1. The mean improvement was sharp between baseline and 6 months, increasing from 1.01 to 3.61, and reaching good fidelity at 12 months (benchmark = 4.0) at 12 months and continuing to increase at 18 months (4.02 and 4.50). The change in IMR fidelity over time was highly significant, *F* (1, 10) = 148.69, p= .00. Post-hoc *t* tests comparing baseline fidelity ratings to 6, 12, and 18 months confirmed sensitivity to change, with *t* values of 7.45, at 6 months, 8.26 at 12 months, and 12.10 at 18 months all significant at p < .001. The standardized mean difference effect size (Cohen’s d_z_) was 3.65.

We also examined change over time looking at the percentage of sites attaining good fidelity (4.0 or higher) at each time period. At baseline, none of the sites had any IMR services whatsoever (10 sites rated 1.0 and one site rated 1.1). The number and percentage of sites attaining good fidelity were 6 (55%) at 6 months, 8 (73%) at 12 months, and 10 (91%) at 18 months. Moreover, the number and percentage attaining very good fidelity (4.5 or higher) were 2 (18%) at 6 months, 6 (55%) at 12 months, and 8 (73%) at 18 months. In summary, most sites attained good fidelity by 6 months and very good fidelity by 12 months.

## Discussion

Overall, the psychometric properties of the IMR Fidelity scale were excellent, with very strong interrater reliability and a high degree of agreement between assessors and very good internal consistency at all three follow-up assessments. The scale was sensitive to change, and the entire rating scale from 1 to 5 was used.

High agreement in the overall decision between the assessors indicates that the scale items are easy to understand and to agree on. Using the entire rating scale from 1 to 5 for all 13 items indicates no restriction of range and being sensitive to change. Using the scale in clinics to document improvement may reinforce good clinical practice. It can also inform the need for specific training in different areas. Because fidelity monitoring leads to understanding and sustainment of practices in the clinics (Bond et al. 2009), the findings reinforce wide use of the IMR Fidelity scale for clinical purposes as well as in research.

In general, research on the psychometric properties of Fidelity scales is lacking (Martinez et al. 2014). This study therefore responds to a strong need. Other Fidelity scales should receive similar psychometric attention.

Our findings identified two items with lower (still adequate) agreement: item seven (IMR goal follow-up) and item 13 (behavioral tailoring for medication). Improving agreement on these items would require written documentation and interviews with patients (Bond et al. 2009).

Seven months after the completion of the formal study, fidelity assessors completed a survey on their experiences using the IMR Fidelity scale. Overall, assessors reported some challenges in finding the relevant data but few other difficulties. They reported that the scale was easy to score and had clear instructions. The assessors perceived that the interviews with practitioners provided the most useful source of information. Interviews with leaders and progress notes were less helpful. Nevertheless, using multiple sources (triangulation) enhances validity.

Although several studies have used the IMR Fidelity scale to measure fidelity, the current study is the first to examine psychometric properties thoroughly. Nonetheless, some limitations deserve mention. The fidelity assessments neither included interviews with patients nor observation of IMR sessions. We have not yet assessed predictive validity, the strongest evidence for utility of a Fidelity scale. Although some studies have shown that core principles predict outcomes (Bartholomew and Kensler 2010; Hasson-Ohayon et al. 2007; McGuire et al. 2012), no published study has thus far examined the predictive validity of the IMR Fidelity scale. Experts recommend regular fidelity monitoring (Bond et al. 2009), which is always difficult to implement (Bond et al. 2014; Egeland et al. 2017; Rychener et al. 2009). The widespread use of Fidelity scales awaits electronic health records designed to facilitate quality measurement.

## Conclusions

The IMR Fidelity scale coheres well, including excellent interrater reliability, internal consistency, sensitivity to change and use of the full scale. Our study supports its use for clinical and research purposes. Other Fidelity scales need similar psychometric evaluations. Widespread use of Fidelity scales will require electronic health records designed to facilitate quality measurement.

## Appendix

The following tables to be included in the Appendix.

See Tables 2, 3.

**Table 2:** Percentage agreement between fidelity assessors on individual items

Item	Description	Baseline (%)	6 months (%)	12 months (%)	18 months (%)	Mean
1	People in session/group	91	100	100	100	98
2	Program length	100	100	100	100	100
3	Curriculum comprehensiveness	100	91	100	82	93
4	Educational handouts	100	100	91	100	98
5	Involvement of significant others	100	100	82	82	91
6	IMR goal-setting	100	100	91	100	98
7	IMR goal follow-up	100	82	73	91	87
8	Motivation-based strategies	100	100	91	91	96
9	Educational techniques	100	100	100	91	98
10	Cognitive-behavioral techniques	100	91	91	91	93
11	Coping skills training	100	91	100	82	93
12	Relapse prevention training	100	82	91	91	91
13	Behavioral tailoring for medication	100	73	82	73	82
	Mean agreement for 13 items	99	93	92	90	94

**Table 3:** Illness Management and Recovery (IMR) Fidelity scale rev. 3-24-05

	1	2	3	4	5
1. *# People in a session or group* IMR is taught individually or in groups of 8 or less consumers	Some sessions taught with over 15 consumers	Some sessions taught with 13–15 consumers	Some sessions taught with 11 or 12 consumers	Some sessions taught with 9 or 10 consumers	All IMR sessions taught individually or in groups of 8 or less
2. *Program length* Consumers receive at least 6 months of weekly IMR sessions or equivalent (e.g., biweekly for at least 12 months)	< 20% of IMR clients receive at least 6 months of weekly sessions	20–39% of IMR clients receive at least 6 months of weekly sessions	40–69% of IMR clients receive at least 6 months of weekly sessions	70–89% of IMR clients receive at least 6 months of weekly sessions	≥ 90% of IMR clients receive at least 6 months of weekly sessions
3. *Comprehensiveness of the curriculum* **•** Recovery strategies • Mental illness facts • Stress-vulnerability model • Social support • Using medication • Preventing relapse • Stress management • Coping symptoms • Mental health system	Curriculum materials include only 1 topic, or educational handouts are not available	Curriculum materials include 2 or 3 topic areas	Curriculum materials include 4 or 5 topic areas	Curriculum materials include 6 or 7 topic areas	Curriculum materials include 8 or 9 topic areas
4. *Provision of educational handouts* All consumers participating in IMR receive IMR handouts	< 20% of IMR clients receive educational handouts	20–39% of IMR clients receive educational handouts	40–69% of IMR clients receive educational handouts	70–89% of IMR clients receive educational handouts	≥ 90% of IMR clients receive educational handouts
5. *Involvement of significant others* At least one IMR-related contact by IMR practitioner in the last month OR significant other has involvement with the consumer in pursuit of goals (e.g., assisting with homework assignments)	< 20% of IMR clients have significant other(s) involved	20–29% of IMR clients have significant other(s) involved	30–39% of IMR clients have significant other(s) involved	40–49% of IMR clients have significant other(s) involved	≥ 50% of IMR clients have significant other(s) involved
6. *IMR goal setting* • Realistic and measurable (ambitious goals are broken down into smaller steps) • Individualized • Pertinent to recovery process • Linked to IMR plan	< 20% of IMR clients have at least 1 personal goal in chart	20–39% of IMR clients have at least 1 personal goal in chart and interviews verify IMR goal setting process	40–69% of IMR clients have at least 1 personal goal in chart and interviews verify IMR goal setting process	70–89% of IMR clients have at least 1 personal goal in chart and interviews verify IMR goal setting process	≥ 90% of IMR clients have at least 1 personal goal in chart and consumer and clinician interviews verify IMR goal setting process
7. *IMR goal follow*-*up* Practitioners and consumers collaboratively follow up on goal(s) (see examples in the IMR Practitioner Workbook)	< 20% of IMR clients have follow-up on goal(s) documented in chart	20–39% of IMR clients have follow-up on goal(s) documented in chart and interviews verify IMR goal follow-up	40–69% of IMR clients have follow-up on goal(s) documented in chart and interviews verify IMR goal follow-up	70–89% of IMR clients have follow-up on goal(s) documented in chart and interviews verify IMR goal follow-up	≥ 90% of IMR clients have follow-up on the goal(s) documented in their chart and consumer and clinician interviews verify IMR goal follow-up
8. *Motivation*-*based strategies* • New info & skills linked to personal goals • Positive perspectives • Pros & cons of change • Hope & self-efficacy	Few or none of the practitioners are familiar with the use of motivation-based strategies (e.g., < 20% of IMR sessions use at least 2 motivation-based strategy)	Some of the practitioners are familiar, with a low level of use (e.g., 20–29% of IMR sessions use at least 2 motivation-based strategy)	Some of the practitioners are familiar, with a moderate level of use (e.g., 30–39% of IMR sessions use at least 2 motivation-based strategy)	The majority of the practitioners are familiar and use it regularly (e.g., 40–49% of IMR sessions use at least 2 motivation-based strategy)	All practitioners are familiar with the use of motivation-based strategies and use it regularly (e.g., ≥ 50% of IMR sessions use at least 2 motivation-based strategy
9. *Educational techniques* • Interactive teaching • Checking for understanding • Breaking down info • Reviewing info	Few or none of the practitioners are familiar with the use of educational strategies (e.g., < 20% of IMR sessions use at least 2 educational strategies)	Some of the practitioners are familiar, with a low level of use (e.g., 20–29% of IMR sessions use at least 2 educational strategies)	Some of the practitioners are familiar, with a moderate level of use (e.g., 30–39% of IMR sessions use at least 1 educational strategies)	The majority of the practitioners are familiar and use it regularly (e.g., 40–49% of IMR sessions use at least 2 educational strategies)	All practitioners are familiar with the use of educational strategies and use it regularly (e.g., ≥50% of IMR sessions use at least 2 educational strategies
10. *Cognitive*-*behavioral techniques* • Reinforcement • Shaping • Modeling • Role playing • Cognitive restructuring • Relaxation training	Few or none of the practitioners are familiar with the use of cognitive-behavioral strategies (e.g., < 20% of IMR sessions use at least 2 cognitive-behavioral strategies)	Some of the practitioners are familiar, with a low level of use (e.g., 20–29% of IMR sessions use at least 2 cognitive-behavioral strategies)	Some of the practitioners are familiar, with a moderate level of use (e.g., 30–39% of IMR sessions use at least 1 cognitive-behavioral strategy)	The majority of the practitioners are familiar and use it regularly (e.g., 40–49% of IMR sessions use at least 2 cognitive-behavioral strategies)	All practitioners are familiar with the use of cognitive-behavioral strategies and use them regularly (e.g., ≥ 50% of IMR sessions use at least 2 cognitive-behavioral strategies
11. *Coping skills training* • Review current coping • Amplify current coping or develop new coping skills • Behavioral rehearsal • Review effectiveness • Modify as necessary	Few or none of the practitioners are familiar with the principles of coping skills training	Some of the practitioners are familiar with the principles of coping skills training, with a low level of use	Some of the practitioners are familiar with the principles of coping skills training, with a moderate level of use	The majority of the practitioners are familiar with the principles of coping skills training and use them regularly	All practitioners are familiar with the principles of coping skills training and use them regularly
12. *Relapse prevention training* • Identify triggers • Identify early warning signs • Stress management • Ongoing monitoring • Rapid intervention as needed	Few or none of the practitioners are familiar with the principles of relapse prevention training	Some of the practitioners are familiar with the principles of relapse prevention training, with a low level of use	Some of the practitioners are familiar with the principles of relapse prevention training, with a moderate level of use	The majority of the practitioners are familiar with the principles of relapse prevention training and use them regularly	All practitioners are familiar with the principles of relapse prevention training and use them regularly, as documented by relapse prevention plans in clients’ charts
13. *Behavioral tailoring for medication* Behavioral tailoring includes developing strategies tailored to each individual’s needs, motives and resources (e.g., choosing medication that requires less frequent dosing, placing medication next to one’s toothbrush)	Few or none of the practitioners are familiar with the principles of behavioral tailoring for medication	Some of the practitioners are familiar with the principles of behavioral tailoring for medication, with a low level of use	Some of the practitioners are familiar with the principles of behavioral tailoring for medication, with a moderate level of use	The majority of the practitioners are familiar with the principles of behavioral tailoring for medication and use them regularly	All practitioners are familiar with the principles of behavioral tailoring for medication and use them regularly

Score all items based on information gathered through interviews with consumers and clinicians, as well as review of IMR documentation in consumers’ charts
